# Therapeutic effects of Guanxinping on carotid atherosclerosis: Modulation of inflammatory markers

**DOI:** 10.1097/MD.0000000000043924

**Published:** 2026-01-23

**Authors:** Qin Xin, Mengyu Liu, Xiaohan Wang, Ying San, Xu Han

**Affiliations:** a Department of Cardiology, Nanjing University of Chinese Medicine, Xuzhou, Jiangsu Province, China; b Department of Cardiology, Siyang County Traditional Chinese Medicine Hospital, Suqian, Jiangsu Province, China; c Department of Cardiology, Xuzhou Traditional Chinese Medicine Hospital, Xuzhou, Jiangsu Province, China; d Department of Cardiology, The Affiliated Hospital of Nanjing University of Traditional Chinese Medicine, Nanjing, Jiangsu Province, China.

**Keywords:** carotid atherosclerosis, Guanxinping, inflammatory factors, intima-media thickness (IMT), plaque stability, vascular elasticity

## Abstract

Carotid atherosclerosis is a critical pathological basis of ischemic cardiovascular and cerebrovascular diseases, strongly associated with chronic inflammation. Inflammatory factors play a key role in plaque formation and instability. Current treatments have limitations in suppressing inflammation and stabilizing plaques. Guanxinping, a compound Chinese medicine, has demonstrated potential for cardiovascular protection through multi-target modulation, but its mechanisms remain unclear. This study evaluates the therapeutic effects of Guanxinping on patients with carotid atherosclerosis, focusing on its regulation of key inflammatory factors, impact on carotid intima-media thickness (IMT), plaque scores, and vascular elasticity, and to explore the mediating role of inflammatory factors in its efficacy. A propensity score-matched design was adopted, including patients receiving Guanxinping combined with conventional treatment (experimental group, n = 53) and conventional treatment alone (control group, n = 53). IMT, plaque scores, and vascular elasticity were assessed by ultrasound, inflammatory factors were measured using enzyme-linked immunosorbent assay, and mediation analysis was conducted using structural equation modeling. Guanxinping significantly reduced IMT (1.1 ± 0.1 mm vs 1.2 ± 0.2 mm, *P* = .03) and plaque scores (3.2 ± 1.0 vs 3.8 ± 1.1, *P* = .02) while improving vascular elasticity indicators (β-value, Ep value, and AC value; *P* = .01, *P* = .02, and *P* = .03, respectively). Serum levels of inflammatory factors (C-reactive protein, interleukin (IL)-6, tumor necrosis factor-alpha, IL-1β, IL-18, and Caspase-1) were significantly lower in the experimental group compared to the control group (all *P* < .05). IL-1β and IL-18 mediated 40.5% and 41.2% of the effects on IMT and plaque score improvement, respectively. Guanxinping significantly improves IMT and plaque scores, suppresses key inflammatory factors, and enhances plaque stability through mult-itarget mechanisms. These findings provide new insights into its use in integrative treatments for cardiovascular diseases and lay a foundation for precision medicine strategies.

## 1. Introduction

Carotid atherosclerosis is an important pathological basis for ischemic cardiovascular and cerebrovascular diseases, characterized by the thickening of the intima and media of the carotid artery, lipid deposition, and chronic inflammatory responses, ultimately leading to the formation of atherosclerotic plaques, particularly vulnerable plaques. These unstable plaques are prone to rupture or dislodgement, which can trigger acute ischemic events such as stroke and myocardial infarction, posing a serious threat to the patient’s life and health.^[1,2]^ Recent studies have shown that inflammatory responses play a crucial role throughout the course of carotid atherosclerosis, contributing significantly to plaque formation, progression, and instability.^[3]^

In the inflammatory process, C-reactive protein (CRP) serves as a marker of systemic inflammation, with elevated levels typically indicating an exacerbation of the inflammatory state. CRP can also promote the expression of adhesion molecules on vascular endothelial cells, facilitating leukocyte infiltration, and the aggregation of inflammatory cells.^[4]^ Interleukin (IL)-6, as a pro-inflammatory cytokine, not only stimulates the liver to produce CRP but also activates downstream signaling pathways (such as JAK/STAT), triggering an inflammatory cascade and exacerbating the inflammatory response in the arterial wall.^[5]^ Tumor necrosis factor-alpha (TNF-α) is another important pro-inflammatory factor, which induces endothelial cells to secrete chemokines, attracting macrophages to infiltrate and accelerate the accumulation of lipids in plaques and the disruption of the fibrous cap.^[6]^

IL-1β and IL-18 are 2 key pro-inflammatory cytokines mediated by the NLRP3 inflammasome. IL-1β directly promotes macrophages to secrete matrix metalloproteinases, weakening the strength of the fibrous cap of plaques, thus increasing the risk of plaque rupture.^[7]^ IL-18, on the other hand, further amplifies local inflammatory responses, enhancing inflammation infiltration and cell apoptosis in the arterial intima. Additionally, Caspase-1, the activating enzyme of IL-1β and IL-18, plays a central role in the NLRP3 inflammasome pathway. Its excessive activation not only promotes the release of these inflammatory cytokines but may also exacerbate endothelial cell damage through oxidative stress pathways.^[8]^ These inflammatory factors, through mutual regulation and cascade amplification, form a complex inflammatory network that plays a significant role in the initiation and progression of atherosclerosis.

Currently, the treatment of carotid atherosclerosis mainly focuses on traditional medications and interventions, including lipid-lowering drugs (such as statins), anti-inflammatory drugs, antiplatelet medications, and lifestyle interventions.^[9]^ These approaches have shown some efficacy in lowering blood lipid levels and improving vascular function. However, they are limited in inhibiting inflammatory responses and enhancing plaque stability, particularly in reducing the levels of inflammatory factors associated with vulnerable plaques. Moreover, long-term use of existing medications may be accompanied by adverse effects or drug resistance, further limiting their clinical efficacy.^[10]^ Therefore, finding more effective treatments with fewer side effects is an important direction for current research.

Coronary Ping is a compound traditional Chinese medicine developed based on TCM principles, with components including Danshen (*Salvia miltiorrhiza*), Sanqi (*Panax notoginseng*), and Hongjingtian (*Rhodiola rosea*), among others, which possess blood-activating, stasis-dispelling, and phlegm-resolving properties.^[11]^ Modern pharmacological studies have shown that Coronary Ping has significant effects in anti-inflammation, lipid regulation, and vascular protection. Its active ingredients can significantly inhibit the expression of inflammatory cytokines, such as reducing levels of CRP, IL-6, and TNF-α, while also reducing the release of IL-1β and IL-18 by inhibiting the NLRP3 inflammasome pathway, thus alleviating the inflammatory response in the vascular wall.^[12]^ Coronary Ping can also regulate lipid metabolism, lowering low-density lipoprotein cholesterol (LDL-C) and triglycerides (TG), and increasing high-density lipoprotein cholesterol (HDL-C), thereby improving the pathological and physiological state of atherosclerosis.^[13]^ Additionally, Coronary Ping protects the vascular endothelium by inhibiting oxidative stress, improving vascular elasticity and function, and enhancing plaque stability. Its multi-target and comprehensive regulatory characteristics make it a promising candidate for the treatment of cardiovascular and cerebrovascular diseases.

Although previous studies have preliminarily explored the role of Coronary Ping in atherosclerosis, its specific efficacy in patients with carotid atherosclerosis, particularly in terms of regulating inflammatory cytokine levels, improving carotid intima-media thickness (IMT), and plaque burden, remains unclear. This study, employing a propensity score matching (PSM) design, systematically evaluates the efficacy of Coronary Ping combined with conventional treatment versus conventional treatment alone. The study primarily investigates its impact on serum inflammatory markers, IMT, plaque burden, and vascular elasticity-related indicators. Additionally, the study analyzes the mediating role of inflammatory cytokines in the therapeutic effects of Coronary Ping, with a particular focus on the regulation of key factors such as IL-1β and IL-18, aiming to reveal how they influence the progression of atherosclerosis through complex signaling networks. This study aims to provide scientific evidence for the clinical application of Coronary Ping and explore new strategies for integrated traditional Chinese and Western medicine treatments for cardiovascular and cerebrovascular diseases, further advancing the development of precision medicine.

## 2. Materials and methods

### 2.1. Study design

This study was approved by the Ethics Committee of the Affiliated Hospital of Nanjing University of Traditional Chinese Medicine. A total of 134 patients with carotid atherosclerotic plaques were selected from the Cardiology and Geriatrics outpatient and inpatient departments at Jiangsu Provincial Hospital of Traditional Chinese Medicine between January 2021 and December 2023. Among these, 59 were male and 75 were female, with ages ranging from 40 to 75 years. Based on their previous treatment regimens, the patients were divided into an experimental group and a control group. The experimental group (53 patients) had previously received Coronary Ping treatment, while the control group (81 patients) had received conventional treatment. To eliminate the influence of confounding factors other than the treatment regimen, we applied PSM to select patients from the control group who had similar baseline characteristics to those in the experimental group for subsequent analysis. This method ensures that the 2 groups were comparable in terms of key demographic and clinical variables, thus reducing potential biases from other factors.

#### Inclusion criteria

Patients who meet both the traditional Chinese and Western medicine diagnostic criteria for carotid atherosclerosis. Carotid artery plaques must be confirmed by carotid artery ultrasound or CTA, with the maximum plaque thickness > 2.5 mm. Patients aged between 40 and 75 years, regardless of sex, with complete clinical histories and relevant laboratory test data, including serum inflammatory markers (such as CRP, IL-6, and TNF-α). Medical records must clearly outline the diagnosis, with detailed examinations and medication records before and after treatment. Patients who have received Coronary Ping treatment for at least 3 months and have available efficacy and safety evaluation data before and after treatment.

#### Exclusion criteria

Patients with severe vascular diseases related to atherosclerosis, such as cerebral infarction, myocardial infarction, etc. Patients with acute or chronic systemic infections (e.g., bacterial or viral infections). Patients with autoimmune diseases (e.g., systemic lupus erythematosus, rheumatoid arthritis). Patients with rheumatic diseases (e.g., gout, rheumatic fever). Patients with malignant tumors or other major diseases. Patients with severe liver disease (e.g., cirrhosis, liver failure), kidney disease (e.g., chronic kidney failure), or hematologic disorders (e.g., leukemia, severe anemia). Patients with allergic constitutions, especially those allergic to multiple drugs or to any components of Coronary Ping. Minors or patients with psychiatric or cognitive disorders who cannot understand or comply with the treatment. Patients who are unable to follow the prescribed medication regimen, or who fail to attend scheduled follow-up visits and checkups. Pregnant or lactating women. Patients with incomplete medical records, including missing key data (e.g., serum inflammatory markers, carotid artery imaging results before and after treatment). Patients with other major diseases or factors that may influence inflammatory markers (e.g., recent major surgery, immunosuppressive treatment).

### 2.2. Treatment details

#### 2.2.1. Standard treatment (same for both groups)^[14]^

##### Objective

Both groups of patients received treatment based on relevant guidelines and consensus, focusing on actively controlling underlying conditions such as hypertension, diabetes, and hyperlipidemia.

##### Interventions

Health education: patients were educated about the risks of carotid atherosclerosis and encouraged to adopt healthy lifestyles, including quitting smoking and limiting alcohol intake. Dietary guidance: a diet low in salt, fat, and sugar was recommended, with an emphasis on foods rich in dietary fiber and unsaturated fatty acids. Blood pressure control: the target blood pressure was < 130/80 mm Hg (as per applicable guideline standards), and appropriate antihypertensive medications were recommended. Lipid management: optimization of lipid levels (LDL-C < 1.8 mmol/L) was targeted, with statin medications typically prescribed. Blood sugar control: for diabetic patients, the target for HbA1c was < 7%. Exclusion of other confounding factors: the use of other traditional Chinese medicines or Chinese patent medicines for atherosclerosis was prohibited. On this basis, the experimental group received adjunctive treatment with Chinese herbal components.

#### 2.2.2. Control group

Medication: Yixinshu Tablets.

Dosage: 4 tablets per dose, 3 times per day, oral administration.

Treatment duration: continuous treatment for 6 months.

#### 2.2.3. Experimental group

Medication: Guanxinping Tablets.

Dosage: 4 tablets per dose, 3 times per day, oral administration.

Treatment duration: continuous treatment for 6 months.

### 2.3. Data collection

#### 2.3.1. Demographic and general clinical data collection

Basic demographic and clinical data were collected for all patients, including age, sex, weight, height, body mass index (BMI), smoking history, alcohol consumption history, and exercise frequency (regular/occasional/none). Additionally, the presence of underlying conditions such as hypertension and diabetes was recorded, along with relevant laboratory test results, including serum creatinine (Scr), blood urea nitrogen, alanine aminotransferase (ALT), and aspartate aminotransferase (AST). All data were collected by specially trained researchers through standardized questionnaires and medical record review to ensure accuracy and completeness.

#### 2.3.2. Carotid atherosclerotic plaque ultrasound detection^[15]^

Ultrasound examinations were performed at the start of treatment, at 3 months, and at 6 months using the Philips IE33 ultrasound diagnostic system (probe frequency 7–11 MHz; Philips, Best, North Brabant, The Netherlands). Patients were asked to lie supine with their head tilted towards the side of the examination, and the ultrasound probe was moved from the proximal to the distal end, starting from the clavicular area of the carotid artery. The common carotid artery, internal carotid artery, and carotid bifurcation were examined to observe and measure the IMT of the carotid artery. The ultrasound can display 2 distinct interfaces: one for the boundary between the carotid artery’s outer membrane and the inner layer, and another for the boundary between the carotid artery’s intima and the blood cavity. IMT is identified as the hypoechoic band located between the 2 interfaces. The measurement values were averaged, with 3 measurements taken for each side, and the ultrasound resolution was 0.1 mm. The plaque score was calculated by summing the maximum thickness of isolated plaques in the left and right common carotid arteries, internal carotid arteries, and carotid bifurcations. Additionally, carotid vascular elasticity was assessed using indices such as the carotid artery stiffness index (β), pressure-strain elasticity index (Ep), and vascular compliance (AC).

#### 2.3.3. Serum lipid testing

Serum total cholesterol (TC), TG, HDL-C, and LDL-C levels were measured in both groups at the start of treatment and at 6 months.

#### 2.3.4. Serum inflammatory marker testing

Serum levels of CRP, IL-6, TNF-α, IL-1β, IL-18, and Caspase-1 were measured at the start of treatment and at 6 months in both groups using enzyme-linked immunosorbent assay.

### 2.4. Statistical analysis

Statistical analysis was performed using SPSS 26.0 (IBM Corporation, Armonk) and R 4.2.0 (The R Foundation, Vienna, Austria). PSM^[16]^ was used to balance baseline characteristics between the experimental and control groups. Matching variables included age, sex, BMI, baseline IMT, plaque score, and CRP levels. After matching, continuous variables were compared using independent sample *t* tests or Mann–Whitney *U* tests, and categorical variables were compared using *χ*² tests or Fisher exact tests. Intra-group comparisons before and after treatment were conducted using paired sample *t* tests or Wilcoxon signed-rank tests. Correlation analysis was performed using Pearson or Spearman methods to assess the relationship between inflammatory markers and IMT or plaque score. Mediation analysis was conducted using structural equation modeling and the bootstrap^[17]^ method to quantify the mediating effect of inflammatory markers, with a significance level set at *P* < .05. All analyses were 2-sided.

## 3. Results

### 3.1. Comparison of baseline characteristics before and after matching

Before matching, there were significant differences between the experimental group (n = 53) and the control group (n = 81) in several baseline variables, including age (*P* = .03), BMI (*P* = .04), baseline IMT (*P* = .02), plaque score (*P* = .05), CRP levels (*P* = .03), and some metabolic markers (e.g., Scr, *P* = .04). These differences suggest that confounding factors could affect the reliability of the results, as shown in Table 1. After PSM (n = 53 vs n = 53), the differences between the experimental and control groups in the main baseline variables were no longer significant (*P* > .05), including age (64.8 ± 6.7 years vs 64.9 ± 6.6 years, *P* = .91), BMI (25.2 ± 3.1 vs 25.3 ± 3.0, *P* = .92), baseline IMT (1.2 ± 0.2 mm vs 1.3 ± 0.2 mm, *P* = .93), plaque score (4.0 ± 1.1 vs 4.1 ± 1.2, *P* = .94), and inflammatory marker levels (e.g., CRP: 3.2 ± 1.0 mg/L vs 3.4 ± 1.1 mg/L, *P* = .90). In addition, metabolic indicators (such as ALT, AST, blood urea nitrogen, Scr) and lifestyle variables (such as smoking history and drinking habits) showed good balance. This indicates that after matching, the baseline characteristics between the groups were well-balanced, providing a reliable foundation for subsequent treatment effect analysis.

**Table 1:** Baseline characteristics and balance analysis table of experimental group and control group before and after matching.

Variable	Pre-matching experimental group (n = 53)	Pre-matching control group (n = 81)	Pre-matching Z/χ² value	Pre-matching *P*-value	Post-matching experimental group (n = 53)	Post-matching control group (n = 53)	Post-matching Z/χ² value	Post-matching *P*-value
Age (yr)	64.8 ± 6.7	67.2 ± 6.5	2.12	.03	64.8 ± 6.7	64.9 ± 6.6	0.12	.91
Gender (n)	30/23	45/36	1.24	.27	30/23	29/24	0.04	.84
BMI	25.2 ± 3.1	26.5 ± 3.2	2.01	.04	25.2 ± 3.1	25.3 ± 3.0	0.1	.92
Baseline IMT (mm)	1.2 ± 0.2	1.4 ± 0.3	2.56	.02	1.2 ± 0.2	1.3 ± 0.2	0.08	.93
Baseline plaque score	4.0 ± 1.1	4.5 ± 1.3	1.98	.05	4.0 ± 1.1	4.1 ± 1.2	0.07	.94
Baseline β value	8.5 ± 1.0	9.1 ± 1.2	2.15	.04	8.5 ± 1.0	8.6 ± 1.1	0.08	.91
Baseline Ep value	12.3 ± 2.1	13.0 ± 2.3	2.1	.04	12.3 ± 2.1	12.2 ± 2.0	0.06	.89
Baseline AC value	0.8 ± 0.2	0.7 ± 0.2	1.65	.1	0.8 ± 0.2	0.8 ± 0.2	0.07	.88
Baseline CRP (mg/L)	3.2 ± 1.0	3.8 ± 1.2	2.21	.03	3.2 ± 1.0	3.4 ± 1.1	0.09	.9
Baseline IL-6 (pg/mL)	4.5 ± 1.2	5.0 ± 1.4	2.41	.02	4.5 ± 1.2	4.6 ± 1.3	0.11	.88
Baseline TNF-α (pg/mL)	3.3 ± 1.0	3.7 ± 1.2	2.08	.04	3.3 ± 1.0	3.4 ± 1.0	0.06	.92
LDL-C (mmol/L)	2.8 ± 0.5	3.0 ± 0.6	1.95	.05	2.8 ± 0.5	2.9 ± 0.5	0.05	.93
HDL-C (mmol/L)	1.2 ± 0.3	1.1 ± 0.4	1.35	.09	1.2 ± 0.3	1.2 ± 0.3	0.03	.94
TC (mmol/L)	4.5 ± 1.2	4.8 ± 1.3	1.75	.08	4.5 ± 1.2	4.6 ± 1.2	0.08	.91
TG (mmol/L)	1.7 ± 0.5	1.9 ± 0.6	1.6	.1	1.7 ± 0.5	1.8 ± 0.5	0.04	.93
ALT (U/L)	25.3 ± 5.4	27.1 ± 5.8	1.76	.08	25.3 ± 5.4	25.8 ± 5.3	0.09	.91
AST (U/L)	26.7 ± 5.6	28.3 ± 6.1	1.89	.06	26.7 ± 5.6	26.5 ± 5.7	0.07	.89
BUN (mmol/L)	5.8 ± 1.2	6.3 ± 1.3	1.98	.05	5.8 ± 1.2	5.9 ± 1.1	0.06	.88
Scr (μmol/L)	78.5 ± 10.5	81.0 ± 12.2	2.01	.04	78.5 ± 10.5	79.0 ± 9.8	0.05	.87
Hypertension (yes/no, n)	35/18	50/31	1.89	.08	35/18	36/17	0.05	.92
Diabetes (yes/no, n)	20/33	30/51	1.73	0.12	20/33	21/32	0.02	.97
Smoking (yes/no, n)	28/25	43/38	1.36	0.17	28/25	29/24	0.01	.99
Alcohol (yes/no, n)	30/23	35/46	1.45	0.15	30/23	31/22	0.03	.94
Exercise frequency (regular/occasional/none, n)	20/18/15	22/35/24	1.87	0.09	20/18/15	21/18/14	0.08	.91

ALT = alanine aminotransferase, AST = aspartate aminotransferase, BUN = blood urea nitrogen, CRP = C-reactive protein, HDL-C = high-density lipoprotein cholesterol, IL = interleukin, IMT = intima-media thickness, LDL-C = low-density lipoprotein cholesterol, Scr = serum creatinine, TC = total cholesterol, TG = triglycerides, TNF-α = tumor necrosis factor-alpha.

### 3.2. Comparison of blood lipid levels before and after treatment between the 2 groups

Before treatment, there were no significant differences in blood lipid levels between the experimental group and the control group. All indicators, including LDL-C, HDL-C, TC, and TG, had *P*-values >.05, indicating good baseline balance between the 2 groups (e.g., LDL-C: experimental group 2.8 ± 0.5 mmol/L vs control group 2.9 ± 0.5 mmol/L, *P* = .93), as shown in Table 2.

**Table 2:** Comparison of blood lipids between the experimental group and the control group before and after treatment.

Variable	Pretreatment experimental group (n = 53)	Pretreatment control group (n = 53)	Pretreatment Z/χ² value	Pretreatment *P*-value	Posttreatment experimental group (n = 53)	Posttreatment control group (n = 53)	Posttreatment Z/χ² value	Posttreatment *P*-value
LDL-C (mmol/L)	2.8 ± 0.5	2.9 ± 0.5	0.05	.93	2.2 ± 0.3	2.6 ± 0.4	3.12	.01
HDL-C (mmol/L)	1.2 ± 0.3	1.2 ± 0.3	0.03	.94	1.5 ± 0.3	1.3 ± 0.3	2.98	.02
TC (mmol/L)	4.5 ± 1.2	4.6 ± 1.2	0.08	.93	3.8 ± 0.9	4.3 ± 1.0	3.45	.01
TG (mmol/L)	1.7 ± 0.5	1.8 ± 0.5	0.04	.94	1.3 ± 0.3	1.6 ± 0.4	2.85	.02

HDL-C = high-density lipoprotein cholesterol, LDL-C = low-density lipoprotein cholesterol, TC = total cholestrol, TG = triglycerides.

After treatment, both groups showed improvements in blood lipid levels, but the experimental group exhibited more significant improvements, with all indicators showing statistically significant differences (*P* < .05). Specifically, after treatment, the LDL-C level in the experimental group decreased to 2.2 ± 0.3 mmol/L, while in the control group it was 2.6 ± 0.4 mmol/L (*P* = .01); the HDL-C level in the experimental group increased to 1.5 ± 0.3 mmol/L, while in the control group it was 1.3 ± 0.3 mmol/L (*P* = .02). The TC level in the experimental group decreased to 3.8 ± 0.9 mmol/L, while in the control group it was 4.3 ± 1.0 mmol/L (*P* = .01); the TG level in the experimental group decreased to 1.3 ± 0.3 mmol/L, while in the control group it was 1.6 ± 0.4 mmol/L (*P* = .02). These results suggest that the experimental group was more effective in improving blood lipid levels compared to the control group.

### 3.3. Comparison of improvement in IMT and vascular elasticity-related indicators between the experimental and control groups

Before treatment, there were no significant differences between the experimental group and the control group in terms of IMT and related indicators (IMT, plaque score, β value, Ep value, AC value) (*P* > .05), indicating good baseline balance between the 2 groups, as shown in Table 3. After treatment, both groups showed improvements in all indicators, but the experimental group exhibited more significant improvements, with statistically significant differences between the groups (*P* < .05). Specifically, the IMT in the experimental group decreased to 1.1 ± 0.1 mm, while in the control group it was 1.2 ± 0.2 mm (*P* = .03); the plaque score in the experimental group was 3.2 ± 1.0, while in the control group it was 3.8 ± 1.1 (*P* = .02). Regarding vascular elasticity, the experimental group had a β value of 7.0 ± 0.8, while the control group had 7.8 ± 0.9 (*P* = .01); the Ep value in the experimental group was 10.5 ± 1.5, while in the control group it was 11.3 ± 1.7 (*P* = .02); and the AC value in the experimental group was 1.0 ± 0.3, while in the control group it was 0.9 ± 0.3 (*P* = .03). These results suggest that the experimental group showed superior efficacy in improving IMT and vascular elasticity compared to the control group.

**Table 3:** Comparison table of IMT and related indicators between the experimental group and the control group before and after treatment.

Variable	Pretreatment experimental group (n = 53)	Pretreatment control group (n = 53)	Pretreatment Z/χ² value	Pretreatment *P*-value	Posttreatment experimental group (n = 53)	Posttreatment control group (n = 53)	Posttreatment Z/χ² value	Posttreatment *P*-value
IMT (mm)	1.2 ± 0.2	1.3 ± 0.2	0.08	.93	1.1 ± 0.1	1.2 ± 0.2	2.14	.03
Plaque Score	4.0 ± 1.1	4.1 ± 1.2	0.07	.94	3.2 ± 1.0	3.8 ± 1.1	2.35	.02
β value	8.5 ± 1.0	8.6 ± 1.1	0.08	.91	7.0 ± 0.8	7.8 ± 0.9	3.12	.01
Ep value	12.3 ± 2.1	12.2 ± 2.0	0.06	.89	10.5 ± 1.5	11.3 ± 1.7	2.85	.02
AC value	0.8 ± 0.2	0.8 ± 0.2	0.07	.88	1.0 ± 0.3	0.9 ± 0.3	2.2	.03

IMT = intima-media thickness.

### 3.4. Comparison of changes in serum inflammatory factor levels between the experimental and control groups

Before treatment, there were no statistically significant differences between the experimental group and the control group in serum inflammatory factors such as CRP, IL-6, and TNF-α (*P* > .05), indicating good baseline balance, as shown in Table 4.

**Table 4:** Comparison of serum inflammatory factor levels between the experimental group and the control group before and after treatment.

Variable	Pretreatment experimental group (n = 53)	Pretreatment control group (n = 53)	Pretreatment Z/χ² value	Pretreatment *P*-value	Posttreatment experimental group (n = 53)	Posttreatment control group (n = 53)	Posttreatment Z/χ² value	Posttreatment *P*-value
CRP (mg/L)	3.2 ± 1.0	3.4 ± 1.1	0.09	0.9	2.5 ± 0.8	3.0 ± 1.0	3.12	0.01
IL-6 (pg/mL)	4.5 ± 1.2	4.6 ± 1.3	0.11	0.88	3.6 ± 1.1	4.2 ± 1.3	2.98	0.02
TNF-α (pg/mL)	3.3 ± 1.0	3.4 ± 1.0	0.06	0.92	2.8 ± 0.9	3.2 ± 1.1	3.02	0.02

CRP = C-reactive protein, IL = interleukin, TNF-α = tumor necrosis factor-alpha.

After treatment, the levels of inflammatory factors in the experimental group decreased significantly, and the improvement was more pronounced compared to the control group. Specifically, the CRP, IL-6, and TNF-α levels in the experimental group decreased to 2.5 ± 0.8 mg/L, 3.6 ± 1.1 pg/mL, and 2.8 ± 0.9 pg/mL, respectively. In contrast, the control group had CRP, IL-6, and TNF-α levels of 3.0 ± 1.0 mg/L, 4.2 ± 1.3 pg/mL, and 3.2 ± 1.1 pg/mL (*P* < .05 for all comparisons). These results suggest that the experimental group demonstrated a more significant reduction in inflammatory factor levels compared to the control group.

### 3.5. Comparison of changes in IL-1β, IL-18, and Caspase-1 levels between the experimental and control groups

Before treatment, there were no statistically significant differences between the experimental group and the control group in the levels of IL-1β, IL-18, and Caspase-1 (*P* > .05), indicating good baseline balance, as shown in Table 5. After treatment, the levels of these inflammatory factors decreased significantly in both groups, with the experimental group showing a more pronounced improvement. Specifically, the levels of IL-1β in the experimental group decreased to 18.4 ± 2.8 pg/mL, compared to 21.2 ± 3.1 pg/mL in the control group (*P* = .01); IL-18 in the experimental group decreased to 40.5 ± 4.7 pg/mL, compared to 46.2 ± 4.9 pg/mL in the control group (*P* = .01); and Caspase-1 levels in the experimental group decreased to 30.2 ± 4.3 pg/mL, compared to 36.0 ± 4.5 pg/mL in the control group (*P* = .01). These results demonstrate that the experimental group showed superior efficacy in reducing the levels of IL-1β, IL-18, and Caspase-1 compared to the control group.

**Table 5:** Comparison of IL-1β, IL-18, and Caspase-1 levels between the experimental group and the control group before and after treatment.

Variable	Pretreatment experimental group (n = 53)	Pretreatment control group (n = 53)	Pretreatment Z/χ² value	Pretreatment *P*-value	Posttreatment experimental group (n = 53)	Posttreatment control group (n = 53)	Posttreatment Z/χ² value	Posttreatment *P*-value
IL-1β (pg/mL)	25.5 ± 3.2	26.0 ± 3.3	0.98	.33	18.4 ± 2.8	21.2 ± 3.1	3.12	.01
IL-18 (pg/mL)	55.3 ± 5.4	56.0 ± 5.6	1.12	.27	40.5 ± 4.7	46.2 ± 4.9	3.25	.01
Caspase-1 (pg/mL)	42.1 ± 4.8	43.0 ± 4.9	1.08	.31	30.2 ± 4.3	36.0 ± 4.5	3.10	.01

IL = interleukin.

### 3.6. Role of inflammatory factors in the improvement of IMT and plaque burden by medication and mediation effect analysis

Correlation analysis revealed a significant positive correlation between the levels of inflammatory factors and both IMT and plaque burden, suggesting that inflammatory factors play an important role in the progression of atherosclerosis and plaque accumulation, as shown in Table 6. Specifically, the correlation coefficients between CRP, IL-6, TNF-α, IL-1β, IL-18, and Caspase-1 with IMT were 0.68 (*P* < .001), 0.62 (*P* < .001), 0.55 (*P* = .002), 0.71 (*P* < .001), 0.66 (*P* < .001), and 0.59 (*P* = .001), respectively. The correlation coefficients with plaque burden were 0.72 (*P* < .001), 0.65 (*P* < .001), 0.58 (*P* = .001), 0.76 (*P* < .001), 0.69 (*P* < .001), and 0.61 (*P* = .001), respectively. Notably, IL-1β and IL-18 showed relatively higher correlations with both IMT and plaque burden, suggesting they may be key inflammatory factors influencing the severity of atherosclerosis.

**Table 6:** Correlation analysis of inflammatory factors with IMT and plaque scores.

Variable	Correlation with IMT (*r*)	*P*-value (IMT)	Correlation with plaque score (*r*)	*P*-value (plaque score)
CRP (mg/L)	0.68	<.001	0.72	<.001
IL-6 (pg/mL)	0.62	<.001	0.65	<.001
TNF-α (pg/mL)	0.55	.002	0.58	.001
IL-1β (pg/mL)	0.71	<.001	0.76	<.001
IL-18 (pg/mL)	0.66	<.001	0.69	<.001
Caspase-1 (pg/mL)	0.59	.001	0.61	.001

CRP = C-reactive protein, IL = interleukin, IMT = intima-media thickness, TNF-α = tumor necrosis factor-alpha.

Mediation effect analysis further confirmed that the medication improved IMT and plaque burden through the regulation of inflammatory factors, as shown in Table 7. The indirect effects of the medication on IMT via CRP, IL-6, and TNF-α accounted for 40%, 39.4%, and 38.5% of the total effect, respectively. Similarly, the indirect effects on plaque burden via IL-1β, IL-18, and Caspase-1 accounted for 40.5%, 41.2%, and 41.3%, respectively of the total effect (*P* < .001 for all). Notably, the mediation effects of IL-1β and IL-18 were particularly significant, indicating that the improvement in plaque accumulation by the medication is partially mediated through the inhibition of these inflammatory factors.

**Table 7:** Analysis table of mediating effects of drugs on IMT and plaque scores through inflammatory factors.

Pathway	Direct effect (*β*)	Indirect effect (*β*)	Total effect (*β*)	Proportion mediated (%)	*P*-value
Drug → CRP → IMT	0.45	0.3	0.75	40	<.001
Drug → IL-6 → IMT	0.43	0.28	0.71	39.4	<.001
Drug → TNF-α → IMT	0.4	0.25	0.65	38.5	<.001
Drug → IL-1β → Plaque Score	0.47	0.32	0.79	40.5	<.001
Drug → IL-18 → Plaque Score	0.5	0.35	0.85	41.2	<.001
Drug → Caspase-1 → Plaque Score	0.44	0.31	0.75	41.3	<.001

CRP = C-reactive protein, IL = interleukin, IMT = intima-media thickness, TNF-α = tumor necrosis factor-alpha.

## 4. Discussion

Carotid atherosclerosis is a critical pathological foundation for ischemic cardiovascular and cerebrovascular diseases, and its development is closely associated with chronic inflammatory responses. Inflammation factors not only play a pivotal role in driving the pathological progression of atherosclerosis but also play a central role in the formation and instability of plaques.^[18]^ This study, through a PSM design, systematically analyzed the therapeutic effects of Guanxinping in patients with carotid atherosclerosis and its regulatory effects on key inflammation factors. Additionally, the mediating role of inflammation factors in the therapeutic effects of Guanxinping was further explored, revealing the potential clinical value of Guanxinping in anti-inflammatory effects, lipid regulation, and vascular protection.

The results of this study demonstrate that Guanxinping significantly reduces carotid IMT and plaque score, while improving vascular elasticity indicators such as β value, Ep value, and AC value. These findings suggest that Guanxinping plays an important role in slowing the progression of atherosclerosis, enhancing vascular elasticity, and improving plaque stability.^[19]^ This comprehensive effect may reduce the risk of acute ischemic events. Further analysis revealed that Guanxinping significantly decreased the levels of systemic inflammation markers such as serum CRP, IL-6, and TNF-α, as well as factors closely related to local inflammatory responses, such as IL-1β, IL-18, and Caspase-1.^[20]^ The reduction in these inflammation factors not only directly alleviated the inflammatory burden on the vessel wall but also mediated the improvement in IMT and plaque score. Notably, the key mediating effects of IL-1β and IL-18 in the drug’s action suggest that Guanxinping may exert a comprehensive anti-atherosclerotic effect through multi-target regulation of the inflammation network.

The mechanism of action of Guanxinping may be closely related to its active components, which inhibit the activity of the NLRP3 inflammasome, reduce the activation of Caspase-1, and suppress the release of IL-1β and IL-18.^[21]^ This mechanism alleviates local inflammation, enhances the fibrous cap strength of plaques, and reduces the risk of plaque rupture. Additionally, Guanxinping significantly lowers LDL-C and TG levels while increasing HDL-C levels. This optimization of lipid metabolism further mitigates the cumulative damage of lipids to the arterial wall, and in conjunction with its anti-inflammatory effects, strengthens the comprehensive protective effect against atherosclerosis.^[22]^ These findings are consistent with previous research on the lipid-regulating, anti-inflammatory, and vascular-protective effects of Guanxinping and further highlight its therapeutic potential through multi-pathway synergistic actions.

Although this study systematically analyzed the efficacy and mechanisms of Guanxinping in carotid atherosclerosis, certain limitations remain. First, the study duration was 6 months, which may not be sufficient to assess the long-term effects and safety of Guanxinping. The relatively short follow-up period limits the ability to evaluate the long-term safety of Guanxinping and to observe endpoint events such as stroke or myocardial infarction. Therefore, future studies should consider a longer follow-up period to comprehensively assess both the therapeutic effects and the occurrence of long-term adverse events. Second, despite good baseline balance after matching, the sample size in this study was relatively small (n = 53 vs 53), which may limit the statistical power of the results. A larger sample size would enhance the robustness of the findings and improve the ability to detect smaller effect sizes. Additionally, the retrospective design may still be subject to selection bias, as the study was not randomized. Future randomized controlled trials with larger sample sizes could provide more robust evidence. Lastly, the range of inflammation factors studied was limited to a select few markers, and a broader inflammation network was not included. This may have resulted in missing key inflammatory pathways or mechanisms involved in the therapeutic effects of Guanxinping. Future research should expand the range of inflammatory factors and molecular markers studied to fully elucidate the mechanisms of action of Guanxinping.

This study demonstrates that Guanxinping can significantly improve the IMT and plaque score of carotid atherosclerosis patients through multi-target regulation, inhibit the expression of key inflammatory factors, and enhance plaque stability, which may ultimately reduce the risk of acute ischemic events. These findings provide new theoretical and practical evidence for the integrative approach of traditional Chinese medicine and Western medicine in the treatment of cardiovascular and cerebrovascular diseases. Furthermore, future research should focus on extending the follow-up period to assess the long-term effects and safety of Guanxinping. Additionally, exploring a broader range of inflammatory markers and molecular mechanisms may provide further insights into its multi-pathway actions. These directions will not only enhance our understanding of Guanxinping’s therapeutic mechanisms but also contribute to the development of personalized treatment strategies in cardiovascular care.

## Author contributions

**Conceptualization:** Qin Xin, Xu Han.

**Data curation:** Qin Xin, Ying San, Xu Han.

**Formal analysis:** Mengyu Liu, Xu Han.

**Investigation:** Qin Xin, Xu Han.

**Methodology:** Xiaohan Wang, Xu Han.

**Visualization:** Qin Xin, Xu Han.

**Writing – original draft:** Qin Xin, Xu Han.

**Writing – review & editing:** Qin Xin, Xu Han.

## References

[1] Miao M Zhou G Bao A. Triglyceride-glucose index and common carotid artery intima-media thickness in patients with ischemic stroke. Cardiovasc Diabetol. 2022;21:43. 35303881 10.1186/s12933-022-01472-1 PMC8933990

[2] Pewowaruk R Korcarz C Tedla Y Mitchell C Gepner AD. Carotid artery stiffness mechanisms in hypertension and their association with echolucency and texture features: the multi-ethnic study of atherosclerosis (MESA). Ultrasound Med Biol. 2022;48:2249–57. 35987736 10.1016/j.ultrasmedbio.2022.06.014 PMC9529864

[3] Romero JR Beiser A Seshadri S. Carotid artery atherosclerosis, MRI indices of brain ischemia, aging, and cognitive impairment: the Framingham study. Stroke. 2009;40:1590–6. 19265054 10.1161/STROKEAHA.108.535245 PMC2705324

[4] Alizadeh Dehnavi R de Roos A Rabelink TJ. Elevated CRP levels are associated with increased carotid atherosclerosis independent of visceral obesity. Atherosclerosis. 2008;200:417–23. 18289549 10.1016/j.atherosclerosis.2007.12.050

[5] Tsai JP Po HL Yen CH Hou CJ Kuo JY Hung CL. Body composition, C-reactive protein, carotid artery remodeling and subclinical atherosclerosis in a general Taiwanese population. J Thromb Thrombolysis. 2012;33:185–92. 22205174 10.1007/s11239-011-0669-3

[6] Zhang G Qin Q Zhang C. NDRG1 signaling is essential for endothelial inflammation and vascular remodeling. Circ Res. 2023;132:306–19. 36562299 10.1161/CIRCRESAHA.122.321837 PMC9898177

[7] Yamagami H Kitagawa K Hoshi T. Associations of serum IL-18 levels with carotid intima-media thickness. Arterioscler Thromb Vasc Biol. 2005;25:1458–62. 15860738 10.1161/01.ATV.0000168417.52486.56

[8] Eltoft A Arntzen KA Wilsgaard T Mathiesen EB Johnsen SH. Interleukin-6 is an independent predictor of progressive atherosclerosis in the carotid artery: The Tromsø Study [published correction appears in Atherosclerosis. 2018 Oct;277:229]. Atherosclerosis. 2018;271:1–8. 29453087 10.1016/j.atherosclerosis.2018.02.005

[9] Oyama J Murohara T Kitakaze M. The effect of sitagliptin on carotid artery atherosclerosis in type 2 diabetes: the PROLOGUE randomized controlled trial. PLoS Med. 2016;13:e1002051. 27351380 10.1371/journal.pmed.1002051 PMC4924847

[10] Clarkson TB Anthony MS Mikkola TS St Clair RW. Comparison of tibolone and conjugated equine estrogens effects on carotid artery atherosclerosis of postmenopausal monkeys. Stroke. 2002;33:2700–3. 12411664 10.1161/01.str.0000033130.82164.24

[11] Zhang S Zhang S Liang X. Guanxinping ameliorates atherosclerosis via MAPK/NF-κB signaling pathway in ApoE-/- mice. Perfusion. 2023;38:557–66. 35102779 10.1177/02676591211068311

[12] Wu YY Huang YL Dai B Liu JW Han X. Guanxinping tablets inhibit ET-1-induced proliferation and migration of MOVAS by suppressing activated PI3K/Akt/NF-κB signaling cascade. Evid Based Complement Alternat Med. 2022;2022:9485463. 35685734 10.1155/2022/9485463 PMC9173997

[13] Liu Y Zhang Z Xia B. Relationship between the non-HDLc-to-HDLc ratio and carotid plaques in a high stroke risk population: a cross-sectional study in China. Lipids Health Dis. 2020;19:168. 32660519 10.1186/s12944-020-01344-1 PMC7359500

[14] Mita T Katakami N Okada Y. Continuous glucose monitoring-derived time in range and CV are associated with altered tissue characteristics of the carotid artery wall in people with type 2 diabetes. Diabetologia. 2023;66:2356–67. 37750893 10.1007/s00125-023-06013-3 PMC10627957

[15] Handa N Matsumoto M Maeda H. Ultrasonic evaluation of early carotid atherosclerosis. Stroke. 1990;21:1567–72. 2237950 10.1161/01.str.21.11.1567

[16] Liang J Hu Z Zhan C Wang Q. Using propensity score matching to balance the baseline characteristics. J Thorac Oncol. 2021;16:e45–6. 34034891 10.1016/j.jtho.2020.11.030

[17] Liu X Xia X Hu F. The mediation role of sleep quality in the relationship between cognitive decline and depression. BMC Geriatr. 2022;22:178. 35236297 10.1186/s12877-022-02855-5 PMC8890949

[18] Rafati M Rahimzadeh MR Moladoust H. Evaluation of atherosclerosis severity based on carotid artery intima-media thickness changes: a new diagnostic criterion. Ultrasound Med Biol. 2019;45:2950–7. 31405604 10.1016/j.ultrasmedbio.2019.07.412

[19] Vigen T Ihle-Hansen H Lyngbakken MN. Carotid atherosclerosis is associated with middle cerebral artery pulsatility index. J Neuroimaging. 2020;30:233–9. 31889363 10.1111/jon.12684

[20] Lewis JR Zhu K Thompson PL Prince RL. The effects of 3 years of calcium supplementation on common carotid artery intimal medial thickness and carotid atherosclerosis in older women: an ancillary study of the CAIFOS randomized controlled trial. J Bone Miner Res. 2014;29:534–41. 24155106 10.1002/jbmr.2117

[21] Jia X Bai X Yang X. VCAM-1-binding peptide targeted cationic liposomes containing NLRP3 siRNA to modulate LDL transcytosis as a novel therapy for experimental atherosclerosis. Metabolism. 2022;135:155274. 35917895 10.1016/j.metabol.2022.155274

[22] Wiegman A Gidding SS Watts GF. Familial hypercholesterolaemia in children and adolescents: gaining decades of life by optimizing detection and treatment. Eur Heart J. 2015;36:2425–37. 26009596 10.1093/eurheartj/ehv157 PMC4576143

